# The Extent of Intracellular Accumulation of Bilirubin Determines Its Anti- or Pro-Oxidant Effect

**DOI:** 10.3390/ijms21218101

**Published:** 2020-10-30

**Authors:** Annalisa Bianco, Aleš Dvořák, Nikola Capková, Camille Gironde, Claudio Tiribelli, Christophe Furger, Libor Vitek, Cristina Bellarosa

**Affiliations:** 1 Italian Liver Foundation (FIF), Bldg Q—AREA Science Park Basovizza, SS14 Km 163,5, 34149 Trieste, Italy; annalisa.bianco@fegato.it (A.B.); ctliver@fegato.it (C.T.); 2 Department of Life Sciences, University of Trieste, 34127 Trieste, Italy; 3 Institute of Medical Biochemistry and Laboratory Diagnostics, Faculty General Hospital and 1st Faculty of Medicine, Charles University, 121 08 Prague 2, Czech Republic; aleshdvorak@gmail.com (A.D.); nikola.capkova@gmail.com (N.C.); vitek@cesnet.cz (L.V.); 4 AOP/MH2F Team, LAAS-CNRS, 7 avenue de l’Europe, 31400 Toulouse, France; cgironde@laas.fr (C.G.); cfurger@laas.fr (C.F.); 5 4^th^ Department of Internal Medicine, Faculty General Hospital and 1^st^ Faculty of Medicine, Charles University, 121 08 Prague 2, Czech Republic

**Keywords:** bilirubin, ROS, antioxidant, redox state, bilirubin neurotoxicity

## Abstract

Background: Severe hyperbilirubinemia can cause permanent neurological damage in particular in neonates, whereas mildly elevated serum bilirubin protects from various oxidative stress-mediated diseases. The present work aimed to establish the intracellular unconjugated bilirubin concentrations (iUCB) thresholds differentiating between anti- and pro-oxidant effects. Methods: Hepatic (HepG2), heart endothelial (H5V), kidney tubular (HK2) and neuronal (SH-SY5Y) cell lines were exposed to increasing concentration of bilirubin. iUCB, cytotoxicity, intracellular reactive oxygen species (ROS) concentrations, and antioxidant capacity (50% efficacy concentration (EC_50_)) were determined. Results: Exposure of SH-SY5Y to UCB concentration > 3.6 µM (iUCB of 25 ng/mg) and >15 µM in H5V and HK2 cells (iUCB of 40 ng/mg) increased intracellular ROS production (*p* < 0.05). EC_50_ of the antioxidant activity was 21 µM (iUCB between 5.4 and 21 ng/mg) in HepG2 cells, 0.68 µM (iUCB between 3.3 and 7.5 ng/mg) in SH-SY5Y cells, 2.4 µM (iUCB between 3 and 6.7 ng/mg) in HK2 cells, and 4 µM (iUCB between 4.7 and 7.5 ng/mg) in H5V cells. Conclusions: In all the cell lines studied, iUCB of around 7 ng/mg protein had antioxidant activities, while iUCB > 25 ng/mg protein resulted in a prooxidant and cytotoxic effects. UCB metabolism was found to be cell-specific resulting in different iUCB.

## 1. Introduction

Unconjugated bilirubin (UCB) is the final product of the heme catabolic pathway in the intravascular compartment. UCB is produced by the activity of heme oxygenase (HMOX), an enzyme that splits the tetrapyrrolic ring of heme into biliverdin, carbon monoxide, and ferrous iron. Subsequently, biliverdin is reduced by biliverdin reductase (BLVR) into UCB, which is transported in blood tightly bound to serum albumin before uptake by the hepatocyte. Only less than 0.1% of UCB is unbound to albumin (so-called free bilirubin, Bf). The Bf fraction determines the biological activities of bilirubin [1].

UCB can diffuse into any cell [2,3] and although being a potent antioxidant at low concentrations, it is toxic at high concentrations. Hence, all cells must maintain the intracellular concentration of UCB below toxic thresholds. This is regulated by its intracellular metabolism (conjugation and oxidation) as well as export out of the cells. UCB interacts mainly with three families of detoxifying enzymes: cytochrome P-450-oxygenase (CYPs) [4,5], glutathione-S-transferases (GSTs) [6], and UDP-glucuronosyltransferases (UGTs) [7]. UCB export is another mechanism used by hepatic and non-hepatic cells to prevent their intracellular accumulation. The export proteins include ATP Binding Cassette Subfamily C Member 2 (ABCC2) involved in the hepatobiliary secretion of bilirubin conjugates, as well as another three ABC transporters that were demonstrated to transport of UCB: ATP Binding Cassette Subfamily B Member 1 (ABCB1 also called MDR1/PGP1) [8], ATP Binding Cassette Subfamily C Member 1 (ABCC1 also called MRP1) [9,10,11,12] and ATP Binding Cassette Subfamily C Member 3 (ABCC3 also called MRP3) [13]. Under physiological conditions, these efflux pumps are expressed in organs involved in the elimination of endo- and xeno-biotics, such as the liver and the kidney, and in epithelial tissues that protect the organs from the entry of xenobiotics, like the small intestine, testes, placenta, and blood–brain barrier (BBB) [14]. 

Hence, bilirubin behavior in a human body has two faces, similar to Janus Bifrons, a Roman god. Elevated serum/plasma UCB concentration, and in particular the Bf fraction, expose babies to the risk of neurotoxicity [15]. Conversely, mildly elevated systemic bilirubin concentrations such as in Gilbert syndrome (GS) [16] protect against various oxidative stress-mediated and metabolic diseases including cardiovascular diseases (CVD), type 2 diabetes, and/or metabolic syndrome [17].

Cells use multiple systems to protect against reactive oxygen species (ROS). Enzymes with antioxidant actions include catalase and superoxide dismutase that together convert superoxide to water. Glutathione (GSH) is regarded as the principal endogenous intracellular small molecule antioxidant cytoprotectant. Studies on cells depleted of GSH or bilirubin indicate that bilirubin is of comparable, or greater, importance to GSH in cytoprotection [18] since bilirubin is one of the most abundant endogenous antioxidants in mammalian tissues [19]. Among extensive series of antioxidants, bilirubin has the most potent superoxide and peroxide radical scavenger activities [20]. The potent physiologic antioxidant actions of bilirubin are further amplified by the oxidation of bilirubin to biliverdin and then recycled by biliverdin reductase back to bilirubin [18].

Nevertheless, it seems that each cell type and tissue may have a different bilirubin threshold switching between beneficial and toxic effects. Doré and Snyder [21] reported that its maximal neuroprotective effects in hippocampal cultures were reached at nanomolar concentrations (10–50 nM), while at higher concentrations the prooxidant effects of bilirubin became dominant. A similar dual effect was reported in the primary cultures of oligodendrocytes, showing protective effects at UCB concentration from 0.05 to 20 µM and a diminished cytoprotective effects at 100 µM [22]. 

However, the exact concentration thresholds between anti- and pro-oxidant effects of bilirubin remain undefined and need further investigation [23]. Thus, in the present work, we performed an in vitro study using different human and murine cell lines exposed to increasing concentrations of UCB, and correlated the intracellular unconjugated bilirubin concentrations with cytotoxic, antioxidant and prooxidant effects.

## 2. Results

### 2.1. UCB Cytotoxicity 

**Figure 1:**
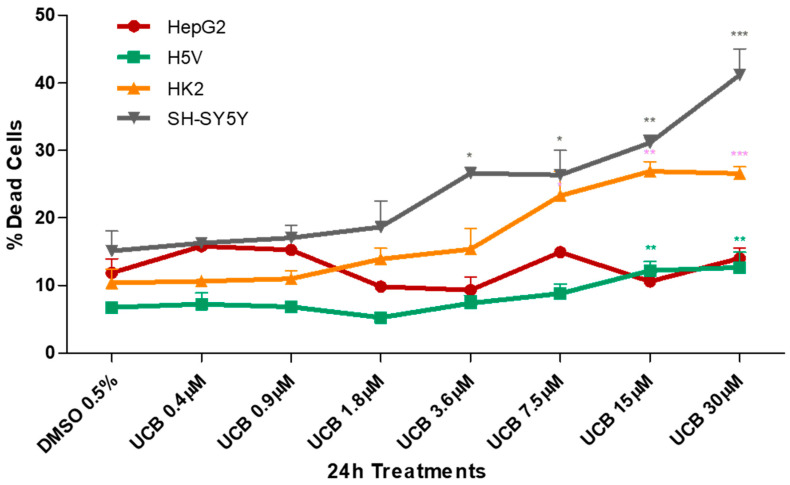
**The effect of unconjugated bilirubin (UCB) treatment on cell viability.** The cell lines were exposed to the increasing UCB concentration (from 0.4 to 30 µM in the presence of 30 µM BSA) or 0.5% DMSO for 24 h and then treated with propidium iodide (PI). The percentage of dead cells was calculated as the proportion of fluorescence intensity of dead cells to that of total cells. Data are expressed as mean ± SD of at least four independent experiments. * *p* < 0.05, ** *p* < 0.01, *** *p* < 0.001).

The four cell lines were exposed to a dose-dependent UCB treatment for 24 h and cytotoxicity was assessed by the propidium iodide (PI) test (Figure 1).

UCB cytotoxicity showed three different levels of susceptibility among the cell lines. HepG2 cell line was less sensitive, while the neuronal cells appeared the most sensitive. HK2 and H5V showed an intermediate dose-dependent cytotoxicity behavior. The first significant increase in dead cells was detected at UCB concentrations of 3.6 µM for SH-SY5Y, 7.5 µM for HK2 and 15 µM for H5V. No changes were observed in HepG2.

**Figure 2:**
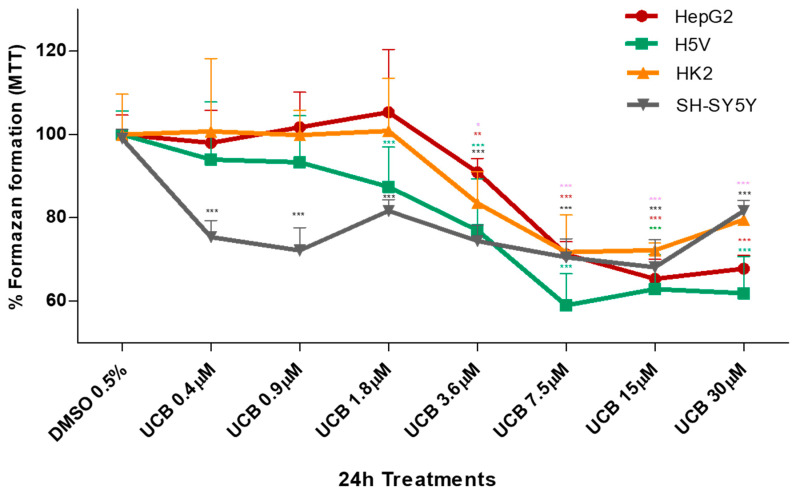
**The effect of UCB on the metabolic activity of studied cells.** The cell lines were exposed to the increasing UCB concentrations (from 0.4 to 30 µM in the presence of 30 µM BSA) or 0.5% DMSO for 24 h and then MTT test was performed. The capacity of control DMSO-treated cells to modify MTT into formazan was considered as 100%. Data are expressed as mean ± SD of at least four independent experiments. * *p* < 0.05, ** *p* < 0.01, *** *p* < 0.00.

The metabolic activity measured by MTT was also substantially affected upon the same UCB exposure (Figure 2). In HepG2 cells, formazan generation was reduced by 30% upon exposure to increasing UCB concentrations, and similar data were obtained also in H5V and HK2 cells, while the SH-SY5Y cells were substantially more sensitive with reduction of formazan generation to 75% upon exposure to as low as 0.4 µM UCB concentrations.

### 2.2. The Effect of UCB Exposure to Intracellular UCB Concentration

All four cell lines were exposed for 24 h to increasing UCB concentrations, and then the intracellular bilirubin concentrations were determined (Table 1).

The intracellular bilirubin concentrations differ substantially among the cell lines, with HepG2 cells being the most resistant (intracellular UCB concentration remained comparable to the control level until 15 µM treatment). SH-SY5Y, HK2, and H5V cells showed a significant dose-dependent intracellular bilirubin content, though the extent differs among the three cell lines: SH-SY5Y was the most sensitive, having one order of magnitude higher intracellular concentrations compared to HepG2 cells (*p* = 0.0014). 

### 2.3. The Effect of UCB Exposure on Intracellular ROS Production

**Figure 3:**
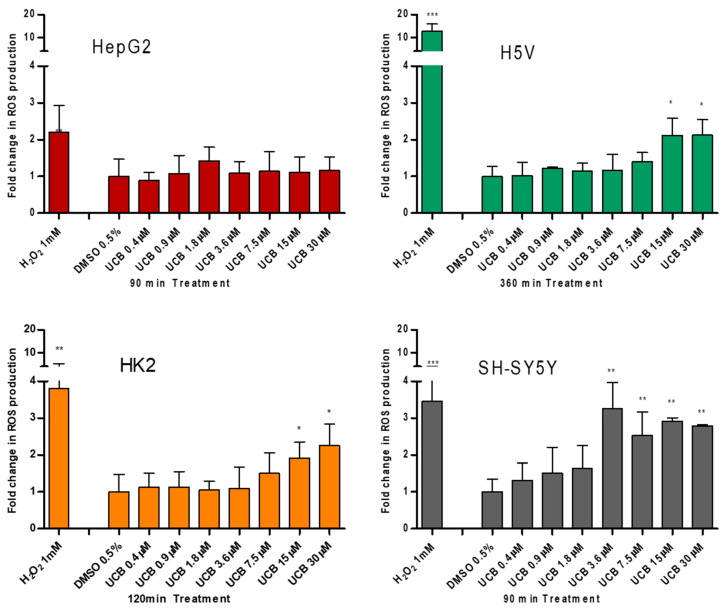
**The effect of UCB treatment on intracellular reactive oxygen species (ROS) production.** The cell lines were exposed to the increasing UCB concentration (from 0.4 to 30 µM in the presence of 30 µM BSA) or 0.5% DMSO for the time indicated on *x*-axis. The time of exposure to UCB was defined based on a time course performed in each cell line. The time with the highest increase in ROS production by UCB and 1 mM H_2_O_2_ (used as positive control) was selected for each cell line (90 min for HepG2 cells, 120 min for SH-SY5Y and HK2 cells, and 360 min for H5V cells). Fluorescence results reflecting ROS production were normalized to the total protein content and compared to DMSO-treated cells. Data are expressed as mean ± SD of three independent experiments. * *p* < 0.05, ** *p* < 0.01, *** *p* < 0.001.

To test the prooxidant ability of UCB, the intracellular ROS production was measured (Figure 3). 

UCB did not result in any significant increase in intracellular ROS production in HepG2 cells at any time point despite a two fold ROS increase by H_2_O_2_ after 45 min (*p* ≤ 0.05) and 90 min (*p* ≤ 0.01) of treatment (Figure S1). On the contrary, in SH-SY5Y cells, UCB concentration higher than 3.6 µM (corresponding to intracellular bilirubin concentration of 25 ng/mg) resulted in a threefold increase in intracellular ROS production. In H5V cells and HK2 cells, UCB treatments higher than 15 µM (corresponding to intracellular bilirubin concentration of 40 ng/mg) doubled the intracellular ROS concentration.

### 2.4. The Antioxidant Effect of UCB on Live Cells Measured by LUCS Technology AOP1 

**Figure 4:**
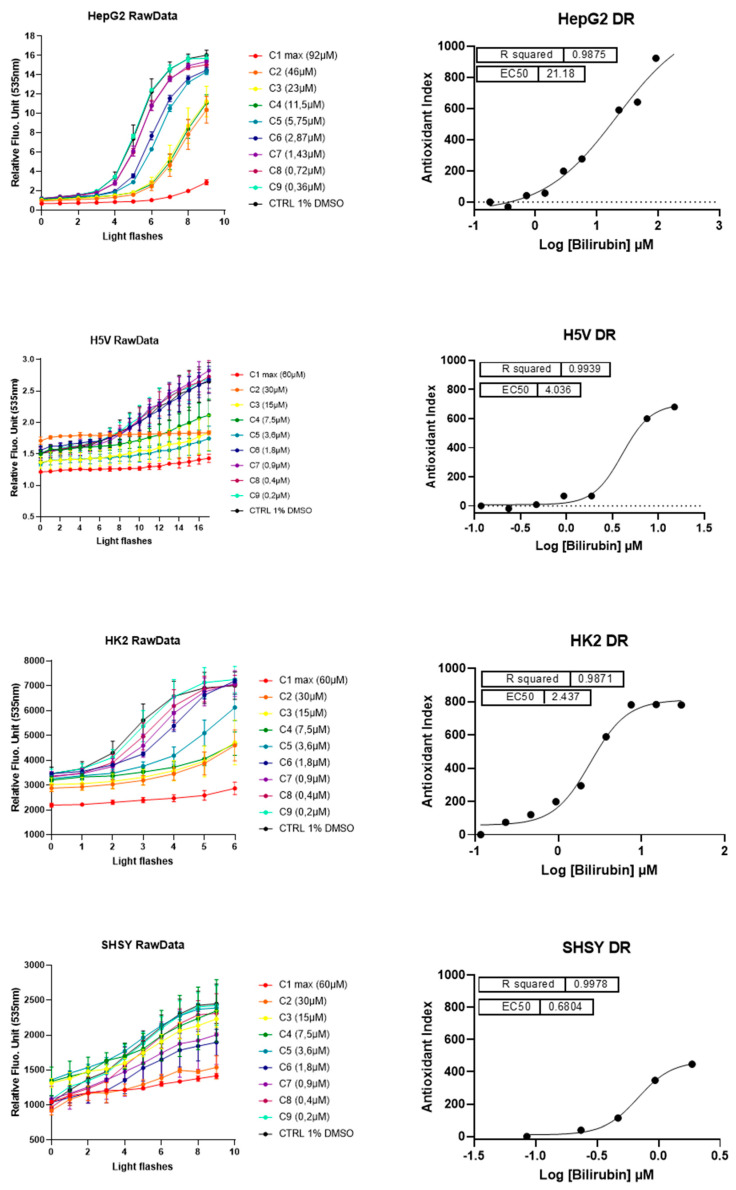
**The antioxidant and cytotoxic effect of UCB**. Each cell line was incubated for 24 h with indicated UCB concentrations and then treated with the fluorescent biosensor for 1 h. Left panel reports the kinetic profiles recorded for each cell line. Relative Fluorescence Units (RFU) were measured at exc/em 501/535 nm according to a recurrent 480 nm LED flash application (20 iterations). Antioxidant effect is measured as a delay (right shift) in fluorescence intensity increase in comparison to negative control profile. Fluorescence intensities higher than negative control at t = 0 (before light application) indicate prooxidant/cytotoxic effects (as describes in [25]). Error bars represent SD values from triplicates. Right panel reports dose–response curves obtained after integration of normalized kinetic data. R^2^s represent determination coefficients obtained by fitting data with a sigmoid regression analysis. Unexpected concentrations C4 (HepG2 cells) and C5 (H5V cells) were removed from the regression analysis. Each series of data corresponds to an experiment representative of at least three experiments.

Having assessed the prooxidant capacity of bilirubin by evaluating intracellular ROS accumulation, we tested possible antioxidant effects of UCB using the Light-Up Cell System (LUCS) technology (Figure 4). LUCS assay measures the ability of a condition to neutralize free radicals produced at the intracellular level by a photo-induction process [24]. When applied on a dose–response mode, the assay allows the evaluation of the 50% efficacy concentration (EC_50_) of the intracellular antioxidant effect of a compound [25].

A direct antioxidant activity of lower concentrations of UCB was detected in all the four cell lines but differ significantly among cell lines. In HepG2 cells, the dose–effect curve showed an EC_50_ of antioxidant effect around 21.2 µM (corresponding to an intracellular UCB concentration of 5.4 ng/mg of total protein). In SH-SY5Y cells, the antioxidant effect showed at 0.68 µM (corresponding to an intracellular UCB concentration between 3.3 and 7.5 ng/mg), whereas in H5V and HK2 cells the antioxidant effect occurred at intermediate UCB concentration. EC_50_ of antioxidant effect was at 4 µM and 2.4 µM, respectively, corresponding to an intracellular UCB concentration between 5 and 7 ng/mg in both cell lines. 

LUCS is a dedicated approach to discriminate between prooxidant/cytotoxic and antioxidant effects [25]. Indeed, prooxidant/cytotoxic effect is revealed at the initial time course by a fluorescence intensity higher than control value [24]. No cytotoxic effect was seen on HepG2 and HK2 cells while it was present at low concentration on SH-SY5Y (treatment of 3.6 µM corresponding to an intracellular UCB concentration of 25 ng/mg) and at very high concentrations (treatment of 30 µM corresponding to an intracellular UCB concentration of 122 ng/mg) on H5V cells. Table 2 reports the intracellular UCB concentration (ng/mg protein) corresponding to the EC_50_ or cytotoxic effect in each cell line. 

### 2.5. The Effect of UCB on Total GSH and SOD Activity 

**Figure 5:**
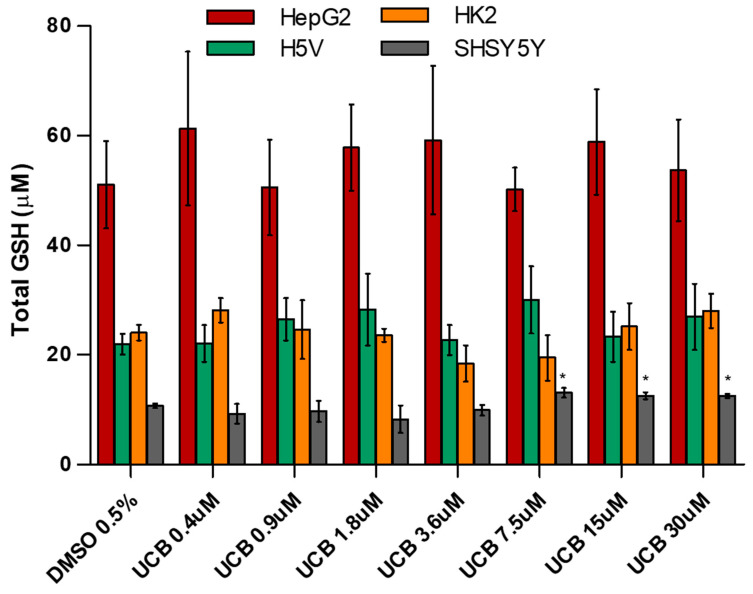
**The effect of UCB exposure on intracellular glutathione (GSH) concentrations.** The cell lines were exposed to increasing UCB concentrations (from 0.4 to 30 µM in the presence of 30 µM BSA) or 0.5% DMSO for 24 h and then total GSH concentration was measured. Data are expressed as mean ± SD of three independent experiments. * *p* < 0.05.

Since the cells use multiple systems to protect against ROS overproduction, we measured total GSH concentrations (Figure 5) and SOD activity (Figure S2) in all the four cell lines exposed for 24 h to increasing UCB concentrations. 

The basal level of GSH was higher in HepG2 cells. GSH concentration was not affected by UCB treatment. The only exception was the SH-SY5Y cells, in which GSH concentrations increased upon a UCB treatment above 7.5 µM (corresponding to an intracellular bilirubin content of 30 ng/mg of total protein). SOD activity was not affected by UCB treatment in HepG2 cells, while its induction was observed in the other cell lines at UCB prooxidant/cytotoxic concentrations.

## 3. Discussion

Severe hyperbilirubinemia can cause permanent neurological damage in neonates [16], while a mild elevation of systemic bilirubin concentrations protects against some diseases such as CVD and diabetes thanks to its antioxidant and anti-inflammatory action [26]. To better understand the determinants of this Janus-like behavior of bilirubin, we performed a comparative study using four different cell lines coming from organs/tissues of different origins. HK2 and H5V derived from normal kidney and heart endothelium and were immortalized by viral transduction (see Methods). HepG2 and SH-SY5Y cells derived from hepatoblastoma and neuroblastoma, respectively. Although they are not normal cells, cancer cell lines are valuable surrogates for in vitro model systems that are widely used in basic and translational research [27] as they provide an unlimited source of biological material. The quantity of cells requested by the experimental plan developed in the present manuscript was not compatible with the yield of primary cell culture. Cells were exposed to the same UCB treatment, the intracellular UCB concentrations were measured and correlated with cytotoxic, antioxidant, and/or prooxidant effects. This approach allowed us to confirm that the intracellular UCB concentrations (and not the external UCB treatment) determine the antioxidant or prooxidant/cytotoxic effects and to define thresholds for antioxidant and cytotoxic effects in these cells.

Intracellular UCB concentration substantially differs among the four cell lines; HepG2 hepatic cells have the lowest concentrations, while the SH-SY5Y neuronal cells are the most sensitive (Table 1). Intracellular UCB concentration depends on several factors including the extent of uptake, excretion, and metabolic transformation, with each of these steps differing in various organs. The ability of HepG2 cells to maintain an intracellular UCB equilibrium in the presence of increasing extracellular UCB treatment is not surprising since the hepatocyte has a flexible and robust system of bilirubin metabolism and detoxification via its conjugation with glucuronic acid by bilirubin UDP-glucuronosyltransferase (UGT1A1) [28,29,30]. In contrast, many cells of different origins do not possess UGT1A1 activity [7] and must either oxidize or export UCB to prevent its intracellular accumulation [14,23]. Hence, the vulnerability of the neurons may be due to lower activities of the mitochondrial enzymes that oxidize UCB as well as the decreased expression of MRP1, one of the bilirubin efflux pump limiting the intracellular accumulation of the pigment [10,11,12,31]. A different pattern of expression of efflux transporters and UCB metabolizing enzymes occurs also on H5V and HK2 cells [32] and can explain the difference of UCB accumulation between hepatic compared to those of non-hepatic origin.

UCB cytotoxicity showed three different levels of susceptibility among the cell lines (Figure 1). As expected, the hepatic HepG2 cell line was less sensitive to UCB toxicity, even at the highest UCB concentration tested (corresponding to an intracellular UCB concentration of 21 ng/mg of protein). Conversely, the neuronal cells appeared the most sensitive since cytotoxicity started at a UCB concentration of 3.6 µM corresponding to an intracellular UCB concentration of 25 ng/mg protein. HK2 and H5V showed an intermediate behavior, with UCB cytotoxicity starting from concentrations of 15 µM corresponding to an intracellular UCB concentration of around 40 ng/mg protein. Our results are consistent with previous data showing that different cells exhibited different susceptibilities to the cytotoxic effects of bilirubin; neuroblastoma was most susceptible while hepatocytes were the least vulnerable [33].

The effect of UCB on cell viability (Figure 1) and metabolic activity (Figure 2) varied substantially. The reduction in the metabolic activity occurred in HepG2 cells at UCB above 3.6 µM but was not associated with the cell toxicity. Similarly, in non-hepatic cell lines, the reduction in formazan formation occurred at UCB concentrations lower than cell mortality (i.e., on SH-SY5Y cells metabolic activity reduction occurred at 0.4 µM while mortality started from 3.6 µM). Most importantly, the percentage of dead cells detected by PI was dose-dependent while the reduction of formazan reached a plateau (25–30% of reduction) at least from UCB treatment of 7.5 µM in all cell lines. UCB has an anti-proliferative activity that prevents the cells from multiplying rapidly and maintains the same number of viable cells [34]. MTT test does not discriminate between cell viability and cell proliferation [35]. The plateau effect seen on MTT test in all cell lines points to the ability of UCB to stop the cell growth. Thanks to this comparative study, we demonstrated that, in case of UCB treatment, MTT test is not a reliable viability test but needs to be supported by a test measuring dead cells.

Among the molecular mechanisms contributing to UCB cytotoxicity, oxidative stress has emerged as a potential crucial event [36]. In various cellular systems, UCB causes ROS production, protein oxidation, and lipid peroxidation [37,38], leading to apoptosis [39]. On the protective side of mild hyperbilirubinemia, in vivo studies suggested that bilirubin significantly reduces the clinical signs of disease where oxidative stress is important, such as in autoimmune encephalomyelitis [22], coronary artery disease [40], renal tubular injury [41] or diabetic nephropathy [42]. Bilirubin has been demonstrated to be a powerful antioxidant substance in in vitro studies [19,20,43], suppressing oxidation more strongly than many other antioxidants, [44,45,46]. Bilirubin at low concentrations exerts its potent cytoprotective effects by bilirubin/biliverdin redox cycling. [18].

The exact concentration thresholds between anti-and prooxidant effects of bilirubin remained undefined [23]. Our results showed that, in spite of ROS induction by H_2_O_2_, the highest UCB treatment dose not induce ROS production in the cell line of hepatic origin (HepG2 cells) while intracellular ROS increase starts in neuronal cell line (SH-SY5Y cells) from UCB concentrations of 3.6 µM, and from 15 µM in both aortic endothelial (H5V) and tubular kidney cells (HK2). On the other side, UCB antioxidant activity showed an EC_50_ of 21.8 µM in HepG2 cells, 0.95 µM in SH-SY5Y cells, 2.44 µM in HK2 cells, and 4 µM in H5V cells. Our results expand and better substantiate what was already published [21,22] showing that each cell type has a different bilirubin threshold switching between the beneficial and toxic effects of bilirubin. Total UCB concentration treatment is an uncertain predictor of its biological effects because intracellular levels of UCB are modulated by its oxidation, conjugation, and export from the cells by membrane ABC transporters [11]. The ability to measure real UCB concentration in the cells much improve our understanding of UCB-induced cytotoxicity as well as its protective effects [3].

**Figure 6:**
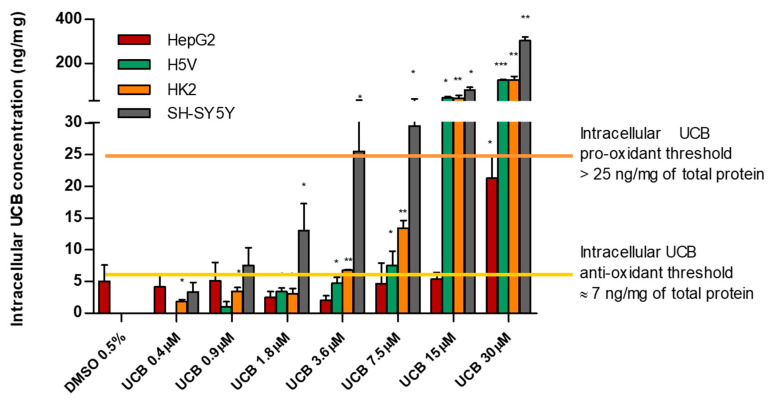
**Intracellular UCB prooxidant and antioxidant thresholds.** The cell lines were exposed for 24 h to the increasing UCB concentration (from 0.4 to 30 µM in the presence of 30 µM BSA) or 0.5% DMSO. Data are expressed as mean ± SD of three independent experiments, UCB concentrations were recalculated per mg of protein. *p*-values represent comparison with control cells. * *p* < 0.05, ** *p* < 0.01, *** *p* < 0.001.

Considering the intracellular UCB concentration regardless of the UCB concentration treatment, we observed that all cell lines have a similar intracellular UCB threshold for antioxidant and prooxidant effects. Figure 6 summarizes all the results previously presented and demonstrates the proposed iUCB threshold. An intracellular UCB concentration around 7 ng/mg acts as an antioxidant, while an intracellular concentration higher than 25 ng/mg is associated with prooxidant and cytotoxic effects.

Finally, we showed that total GSH concentrations and SOD activity, other cellular systems to protect against ROS, do not contribute to the UCB antioxidant activity. Total GSH concentrations (Figure 5) are not influenced by UCB treatment, maintaining the same level of control cells upon UCB dose-dependent treatment. The only exception is on SH-SY5Y cells where GSH levels increased significantly upon a UCB treatment higher than 7.5 µM (corresponding to an intracellular bilirubin content of 30 ng/mg of total protein). These results confirmed what was previously observed by our group when UCB modulated the GSH concentration in neuroblastoma cells through the induction of the System Xc- increasing cysteine uptake and intracellular GSH content [47]. In addition, SOD activity (Figure S2) increased in all cell lines in response to UCB’s prooxidant effect [48].

## 4. Materials and Methods

### 4.1. Cell Cultures

SH-SY5Y human neuroblastoma cells (ATCC-CRL-2266) were maintained in EMEM/F12 1:1 supplemented with 15% fetal bovine serum (FBS), 1% penicillin/streptomycin solution (penicillin G (100 U/mL), streptomycin (100 mg/mL)), l-glutamine (2 mmol/L) (Euroclone S.p.A., Milano, Italy) and 1% non-essential amino acids (Sigma-Aldrich, St Louis, MO, USA).

HepG2 human hepatoblastoma cells were maintained in DMEM high glucose supplemented with 10% FBS, 1% penicillin/streptomycin solution (penicillin G (100 U/mL), streptomycin (100 mg/mL)), l-glutamine (2 mmol/L).

H5V, murine heart endothelial cells transformed by polyomavirus middle T antigen (kindly provided by Istituto Mario Negri, Milan, Italy), were grown in DMEM low glucose containing 10% (*v*/*v*) FBS, 1% penicillin/streptomycin solution (penicillin G (100 U/mL), streptomycin (100 mg/mL)), l-glutamine (2 mmol/L).

HK2, papillomavirus 16 transformed human proximal tubular epithelial cell line (kindly provided by Prof. R. Bulla, Department of Life Sciences, University of Trieste), was cultured in DMEM low glucose, Ham’s F12 media (1:1) supplemented with decomplemented 5% (*v*/*v*) FBS, 1% penicillin/streptomycin solution (penicillin G (100 U/mL)), streptomycin (100 mg/mL)), l-glutamine (2 mmol/L), bovine insulin (5 mg/mL), holo-transferrin (5 mg/mL), sodium selenite (5 ng/mL), hydrocortisone (5 ng/mL), EGF (10 ng/mL), T3 (5 pg/mL), and PGE (15 pg/mL).

When 80% of confluence was achieved, the cells were used in studies as described below.

### 4.2. Treatments

UCB (Sigma-Aldrich, St Louis, MO, USA) was purified as previously described [49], dissolved in DMSO (6 mM), and added to the cell medium (completed with FBS 10% and BSA in order to achieve a final BSA concentration of 30 µM) to reach a range of final concentration from 0.4 to 30 µM performing serial dilution. DMSO (0.5%) was used to treat control cells.

### 4.3. Quantification of Intracellular UCB Concentration

The cells at 80 % confluence were incubated with different UCB concentrations (from 0.4 to 30 µM) for 24 h, or 0.5 % DMSO. After treatment time, the cells were collected by centrifugation and washed three times in PBS. The intracellular UCB level was quantified using an LC-MS/MS method as was described previously [50].

Briefly, the cells were mixed with internal standard (mesobilirubin), then lysed and deproteinated by 0.5% ammonium acetate in methanol. Finally, this suspension was sonicated and centrifuged. After the final centrifugation steps, 100 μL of supernatant was pipetted into glass vials with the inert insert (suitable for liquid chromatography-tandem mass spectrometry (LC-MS) analysis), and 2 μL was directly injected into the LC-MS apparatus. The LC-MS/MS analyses were performed using high-performance liquid chromatography (Dionex Ultimate 3000, Dionex Softron GmbH, Germany) equipped with a Poroshell 120 EC-C18 column (2.1 μm, 3.0 × 100 mm; Agilent, CA, USA). For a gradient elution, the phase was prepared by mixing 1 mM of NH_4_F (Honeywell, International Inc., Morris Plains, NJ, USA) in water and methanol (40% → 100%, 0–13 min) (Biosolve Chimie SARL, Dieuze, France) [50]. The analytes were detected by mass spectrometer (TSQ Quantum Access Max with a HESI-II probe, Thermo Fisher Scientific, Waltham, MA, USA) operating in a positive SRM mode: bilirubin [585.3 → 299.1 (20 V); 585.3 → 271.2 (18 V)]; MBR [589.3 → 301.1 (20 V); 589.3 → 273.2 (44 V)]. Protein concentration in the cell suspension was determined by DC Protein Assay (Bio-Rad Laboratories, Irvine, CA, USA) and results were expressed as nmol/mg of protein. All steps were performed at dim light.

### 4.4. Cytotoxicity Test (PI) and Metabolic Activity Test (MTT)

The tests were performed on cells cultured in black 96-well plates [51,52].

PI (Sigma-Aldrich, St Louis, MO, USA) was solubilized in PBS to a final concentration of 50 µg/mL. After 60 min of incubation at 37 °C, the initial fluorescence intensity from the dead cells was measured using a multiplate reader (EnSpire 2300, PerkinElmer, Waltham, MA, USA). The excitation and the emission wavelengths were 530 and 620 nm, respectively. After the initial intensity was obtained, Triton X-100 at a final concentration of 0.6% was added to each well to permeabilize the cells and label all nuclei with PI. After 30 min of incubation on ice, fluorescence intensity was re-measured to obtain a value corresponding to the total cells. The percentage of dead cells was calculated as the proportion of fluorescence intensity of dead cells to that of total cells.

Metabolic activity was determined by assessing the reduction of 3(4,5-dimethyl thiazolyl-2)-2,5 diphenyl tetrazolium (MTT, Sigma-Aldrich, St Louis, MO, USA) to formazan by succinate dehydrogenase, a mitochondrial enzyme, as previously described. In brief, a stock solution of MTT was prepared in PBS (5 mg/mL). MTT solution was diluted to 0.5 mg/mL in the cell medium. The test was performed on the cells cultured in 96-well plates. Cells were incubated with the cell medium containing MTT for 1 h at 37 °C. At the end of the incubation period, the insoluble formazan crystals were dissolved in 100 µL of DMSO. Absorbance was determined at 562 nm using a multiplate reader (EnSpire 2300, PerkinElmer, Waltham, MA, USA). Results were expressed as the percentage of control cells not exposed to UCB, which were considered as being 100% viable. Is it worth noting that MTT test is a quantitative colorimetric assay for detecting living, but not dead, mammalian cell survival and proliferation [53].

### 4.5. Determination of Intracellular ROS Production

Intracellular ROS concentrations were monitored by using the fluorescent dye 2′7′-dichlorodihydrofluorescein diacetate (H_2_DCFDA, Invitrogen, Thermo Fisher Scientific, Waltham, MA, USA), which is a non-polar compound converted into a non-fluorescent polar derivative (H2DCF) by cellular esterases after incorporation into cells. H2DCF is membrane-impermeable and is oxidized rapidly to the highly fluorescent 2′,7-dichlorofluorescein (DCF) in the presence of intracellular ROS. Cells were seeded in 96-well black plates. At the end of UCB treatments, the cells were washed with PBS and were post-treated for 1 h with 10 µM H2DCF-DA diluted in serum-free medium (free phenol red-DMEM high glucose 24.5 mM) (Sigma-Aldrich, St Louis, MO, USA). H_2_O_2_ treatment was used as a positive control. Cells were washed in PBS and then incubated in PBS solution to read a normal fluorescence (EnSpire 2300, PerkinElmer, Waltham, MA, USA) at an excitation and emission wavelengths of 505 and 525 nm, respectively. Time of exposure to UCB was defined by a time course performed in each cell line. The time with the highest peak of ROS increase, both by UCB and by H_2_O_2_ 1 mM (used as positive control), was chosen for each cell line (90 min for HepG2 cells, 120 min for SH-SY5Y and HK2 cells, and 360 min for H5V cells).

### 4.6. Anti Oxidant Power 1 (AOP1)

HepG2, SHSY5Y, H5V, and HK2 cells were seeded in 96-well plates and then incubated with UCB (9 concentrations obtained by serial log2 dilutions) in the cell growth medium in the presence of FBS plus BSA to reach BSA final concentration of 30µM. Cells were treated for 24 h at 37 °C in the presence of 5% CO_2_. At least three independent experiments were performed each on triplicate wells. LUCS assay measures the ability of an antioxidant to neutralize oxidative stress and the effect is measured by a delay in the kinetic evolution of fluorescence emission according to Gironde et al. [25]. Briefly, after the 24-h incubation, the cells were treated with the fluorescent biosensor thiazole orange (4 µM, Sigma-Aldrich, St Louis, MO, USA) for 1 h and Relative Fluorescence Units (RFU) at 535 nm were recorded (Varioskan, Thermo Fisher Scientific, Waltham, MA, USA) after a recurrent (20 iterations) 480 nm LED application procedure (24 mJ/cm^2^) [25] of the whole 96-well plate. Kinetic profiles were recorded and dose–response curves were calculated. Prooxidant/cytotoxic effect is revealed at the initial time course by a fluorescence intensity higher than control value [24]. RFUs (Relative Fluorescence Units) presented in Figure 4 were plotted in a kinetics-like mode and analyzed by Prism8 software (GraphPad, San Diego, CA, USA) to generate dose–response curves. Results were normalized to control data and expressed as a Cellular Antioxidant Index (CAI) corresponding to the integration of all normalized data following the equation CAI = 1000 − 1000*(AUCx/AUCcontrol) where AUCx = 0∫12 NFUFNx and AUCcontrol = 0∫12 NFUFNcontrol and NFUFNx = flash number x. For dose–response experiments, CAI values were then used to calculate 50% efficacy concentration (EC50) values from a mathematical non-linear regression model (sigmoid fit) following the equation: Y  =  Bottom  +  (Top − Bottom)/(1  +  10^(LogEC50 − X)*HillSlope^), where HillSlope = slope coefficient of the tangent at the inflection point. EC50 and R2 values were deduced from the regression model. Two AUCs whose value was above AUC values obtained from higher concentrations were discarded from the fit model as regression sigmoid model should describe an increasing function.

### 4.7. GSH Determinations

Total GSH concentration was determined using a GSH Colorimetric Detection Kit (Invitrogen, Thermo Fisher Scientific, Waltham, MA, USA). The kit uses a colorimetric substrate that reacts with the free thiol group on GSH to produce a highly colored product. The cells were cultured in a 6-well plate at different concentrations according to the cell size. When a confluence of around 80% was reached, the cells were treated with different bilirubin concentrations. Afterward, the cells were washed with ice-cold PBS, suspended in ice-cold 5% aqueous 5-sulfosalicylic acid dehydrate, and sonicated to lyse cells. The dilution and assay were conducted as indicated by the kit instructions (the end-point method). Total GSH concentrations (µM) were obtained by interpolation on the standard curve. Results were normalized per 100,000 seeded cells.

### 4.8. SOD Activity

The superoxide dismutase activity was measured according to Ewing and Janero (1995) [54]. SOD activity was measured using an NBT/NADH/PMS system. The non-enzymatic phenazine methosulfate-nicotinamide adenine dinucleotide (PMS/NADH) system generates superoxide radicals that reduce nitro blue tetrazolium (NBT) into a purple-colored formazan. One SOD activity unit was defined as the amount of an enzyme required to cause 50% inhibition of the NBT photoreduction rate. Cell lysates were added to a reaction mixture containing 50 mM potassium phosphate, pH 7.0, 166 µM NADH, 43 µM NBT. The reaction was initiated with the addition of 50 µL freshly prepared PMS 0.75 µM. The absorbance (considered as an index of NBT reduction) was monitored at 560 nm over 5 min every 30 min using a multiplate reader (EnSpire 2300, PerkinElmer, Waltham, MA, USA) in the kinetic mode. The change in absorbance at 560 nm was plotted as a function of time. The slope obtained in the absence of SOD (the activity control) should be maximal and is taken as the 100% value; all other slopes generated with SOD standards or cell tissue extracts were compared to this slope. The % inhibition of the rate of increase in absorbance at 560 nm is calculated as follows: % Inhibition = ((Slope of 1X SOD Buffer Control − Slope of Sample) × 100)/Slope of 1XSOD Buffer Control.

IC50 values of the samples were determined by plotting percentage inhibition vs. the quantity (mg of total proteins) of the cell extract. The SOD activity was expressed in terms of units/mg of total protein considering that one SOD activity unit was defined as the amount of enzyme required to cause 50% inhibition of the NBT photoreduction rate. Protein concentration was determined by Bicinchoninic Acid Protein assay (Sigma-Aldrich, St Louis, MO, USA). Bovine SOD Cu,Zn-SOD (SOD1) (Sigma-Aldrich, St Louis, MO, USA) was used as an internal control to generate a standard curve of the SOD activity.

### 4.9. Statistical Analysis

All data are presented as mean ± standard deviation. A statistically significant difference between two data sets was assessed by unpaired two-tailed Student’s t-test using Prism5 software (GraphPad, San Diego, CA, USA). Statistical significance was determined at *p* < 0.05, unless otherwise indicated. Significance was graphically indicated as follows: * *p* < 0.05, ** *p* < 0.01, *** *p* < 0.001.

## 5. Conclusions

In all the cell lines studied, the intracellular UCB concentration of around 7 ng/mg protein had antioxidant activities, while its intracellular concentrations > 25 ng/mg protein resulted in prooxidant and cytotoxic effects. UCB metabolism was found to be cell-specific resulting in different UCB intracellular concentrations. Nevertheless, we could define the threshold of intracellular UCB concentration valid for various cell types that set the switch between the anti- and pro-oxidant effects of bilirubin.

## Figures and Tables

**Table 1:** **The effect of UCB exposure to intracellular UCB concentrations.** The cell lines were exposed for 24 h to the increasing UCB concentrations (from 0.4 to 30 µM in the presence of 30 µM BSA) or 0.5% DMSO. Data are expressed as mean ± SD of three independent experiments, UCB concentrations were recalculated per mg of protein. *p*-values represent comparison with control cells.

	HepG2		H5V		HK2		SH-SY5Y	
Treatment	Mean ± SD (ng/mg)	*p*-Value (vs. Ctrl)	Mean ± SD (ng/mg)	*p*-Value (vs. Ctrl)	Mean ± SD (ng/mg)	*p*-Value (vs. Ctrl)	Mean ± SD (ng/mg)	*p*-Value (vs. Ctrl)
**Control**	5.0 ± 1.8		0.0 ± 0.0		0.0 ± 0.0		0.0 ± 0.0	
**UCB 0.4 µM**	4.1 ± 1.5	0.735	0.0 ± 0.0		1.8 ± 0.2	0.014	3.34 ± 1.06	0.503
**UCB 0.9 µM**	5.1 ± 2.0	0.984	1 ± 1.09	0.546	3.5 ± 0.4	0.013	7.5 ± 2.0	0.309
**UCB 1.8 µM**	2.4 ± 0.7	0.311	3.4 ± 3.51	0.415	3.0 ± 0.6	0.037	13.0 ± 3.0	0.163
**UCB 3.6 µM**	2.0 ± 0.5	0.245	4.7 ± 0.6	0.018	6.7 ± 0.1	0.000	25.5 ± 5.4	0.043
**UCB 7.5 µM**	4.6 ± 2.3	0.900	7.5 ± 1.6	0.042	13.4 ± 0.9	0.004	29.5 ± 6.3	0.043
**UCB 15 µM**	5.4 ± 0.7	0.874	42.4 ± 4.5	0.011	40.1 ± 9.5	0.052	79.1 ± 8.4	0.011
**UCB 30 µM**	21.3 ± 2.4	0.033	122.3 ± 2.8	0.001	123.8 ± 11.5	0.009	303.3 ± 11.2	0.001

**Table 2:** The intracellular UCB concentrations corresponding to antioxidant (50% efficacy concentration (EC_50_)) or cytotoxic effects.

Cell Lines	Antioxidant EC_50_		Cytotoxic Effect	
	UCB Treatment	Intracellular UCB Content (ng/mg Total Protein)	UCB Treatment	Intracellular UCB Content (ng/mg Total Protein)
**HepG2**	21.2 µM	between 5.4 and 21.3 ng/mg total protein		
**H5V**	4.04 µM	between 4.7 and 7.5 ng/mg total protein	>30 µM	>122.3 ng/mg
**HK2**	2.44 µM	between 3 and 6.7 ng/mg total protein		
**SH-SY5Y**	0.68 µM	between 3.3 and 7.5 ng/mg total protein	>3.6 µM	>25.5 ng/mg

## Supplementary Materials

Supplementary materials can be found at https://www.mdpi.com/1422-0067/21/21/8101/s1.

## Abbreviations

iUCB	Intracellular unconjugated bilirubin concentrations
UCB	Unconjugated bilirubin
EC50	Half maximal effective concentration
ROS	Reactive oxygen species
HMOX	Heme oxygenase
BLVR	Biliverdin reductase
Bf	Free bilirubin
CYPs	Cytochrome P-450-oxygenases
GSTs	Glutathione S-transferases
UGTs	UDP-Glucuronosyl-transferase
ABC	ATP Binding Cassette
ABCC1	ATP Binding Cassette Subfamily C Member 1
ABCC2	ATP Binding Cassette Subfamily C Member 2
ABCC3	ATP Binding Cassette Subfamily C Member 3
ABCB1	ATP Binding Cassette Subfamily B Member 1
MDR1	Multidrug resistance protein 1
MRP3	Multidrug resistance-associated Protein 3
PGP1	P-glycoprotein 1
BBB	Blood–brain barrier
GS	Gilbert syndrome
CVD	Cardiovascular diseases
PI	Propidium iodide
DMSO	Dimethyl sulfoxide
MTT	3(4,5-dimethylthiazolyl-2)-2,5 diphenyl tetrazolium
H2DCFDA	2′7′-dichlorodihydrofluorescein diacetate
DCF	2′,7-dichlorofluorescein
H_2_O_2_	Hydrogen peroxide
BSA	Bovine serum albumin
LUCs	Light-Up Cell System
AOP	Antioxidant powerl
RFU	Relative fluorescence units
SOD	Superoxide dismutase
UGT1A1	UDP Glucuronosyltransferase family 1 member A1
GSH	Reduced glutathione
NBT	Nitro blue tetrazolium
NADH	β-Nicotinamide adenine dinucleotide reduced
PMS	Phenazine methosulfate
CuZnSOD	Copper- and zinc-containing superoxide dismutase
SOD1	Superoxide dismutase [Cu-Zn]
