# Supplementary material for: The Extent of Intracellular Accumulation of Bilirubin Determines Its Anti- or Pro-Oxidant Effect
Source: Int J Mol Sci. 2020 Oct 30;21(21):8101. doi: 10.3390/ijms21218101 (PMC7663266; doi:10.3390/ijms21218101)
Supplement: Supplementary file 1 [file ijms-21-08101-s001.pdf]

International Journal of
Molecular Sciences

MDPI

Supplementary Materials

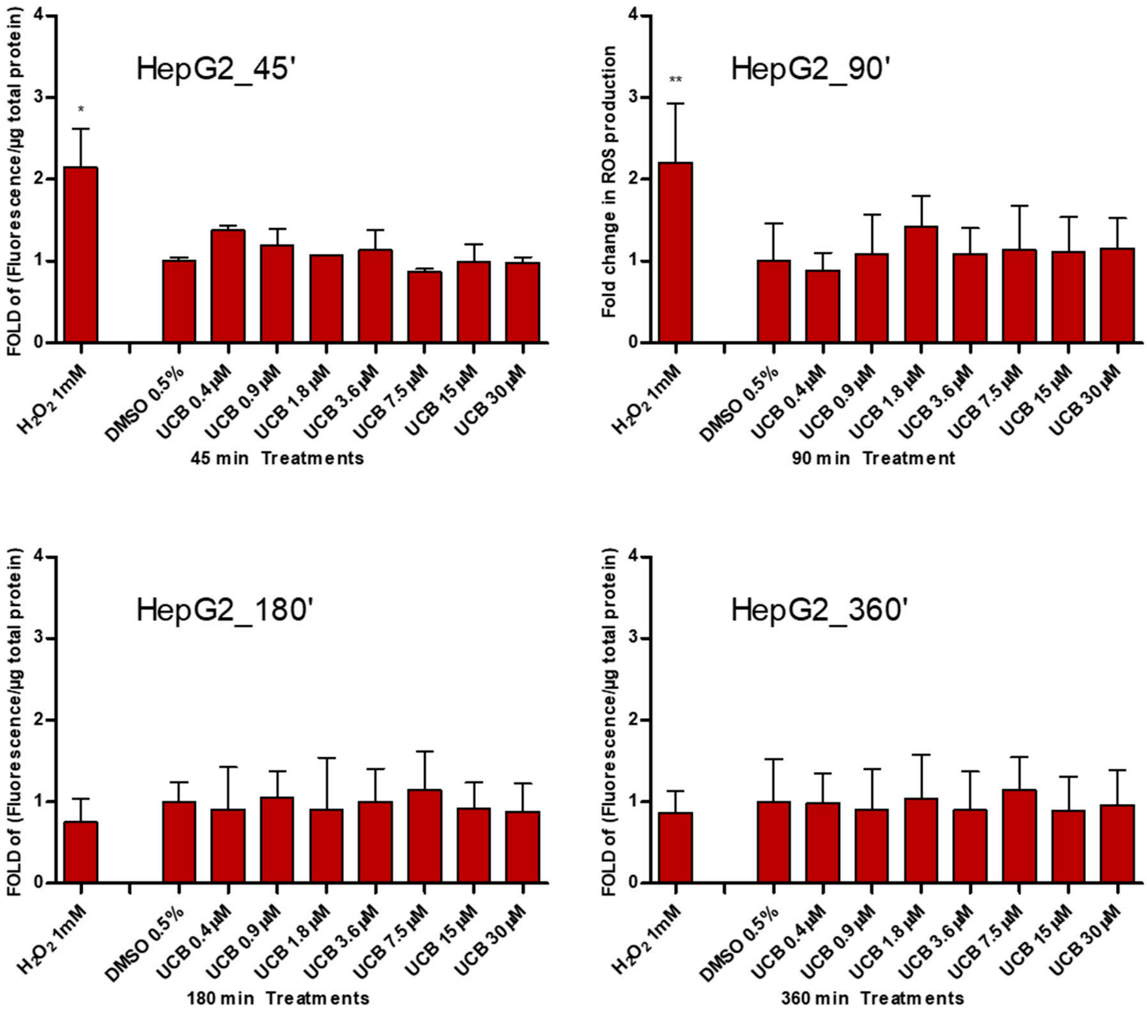

Figure S1. Time course of the effect of UCB treatment on intracellular ROS production in HepG2 cells. HepG2 cells were exposed to increasing UCB concentration (from 0.4 to 30 μM in the presence of 30 μM BSA) or 0.5% DMSO for the time indicated on x-axis. Exposure to 1 mM H_2O_2 was used as positive control. Fluorescence results reflecting ROS production were normalized the total protein content and compared to DMSO-treated cells. Data are expressed as mean±SD of three independent experiments. **p*<0.05, ***p*<0.01.

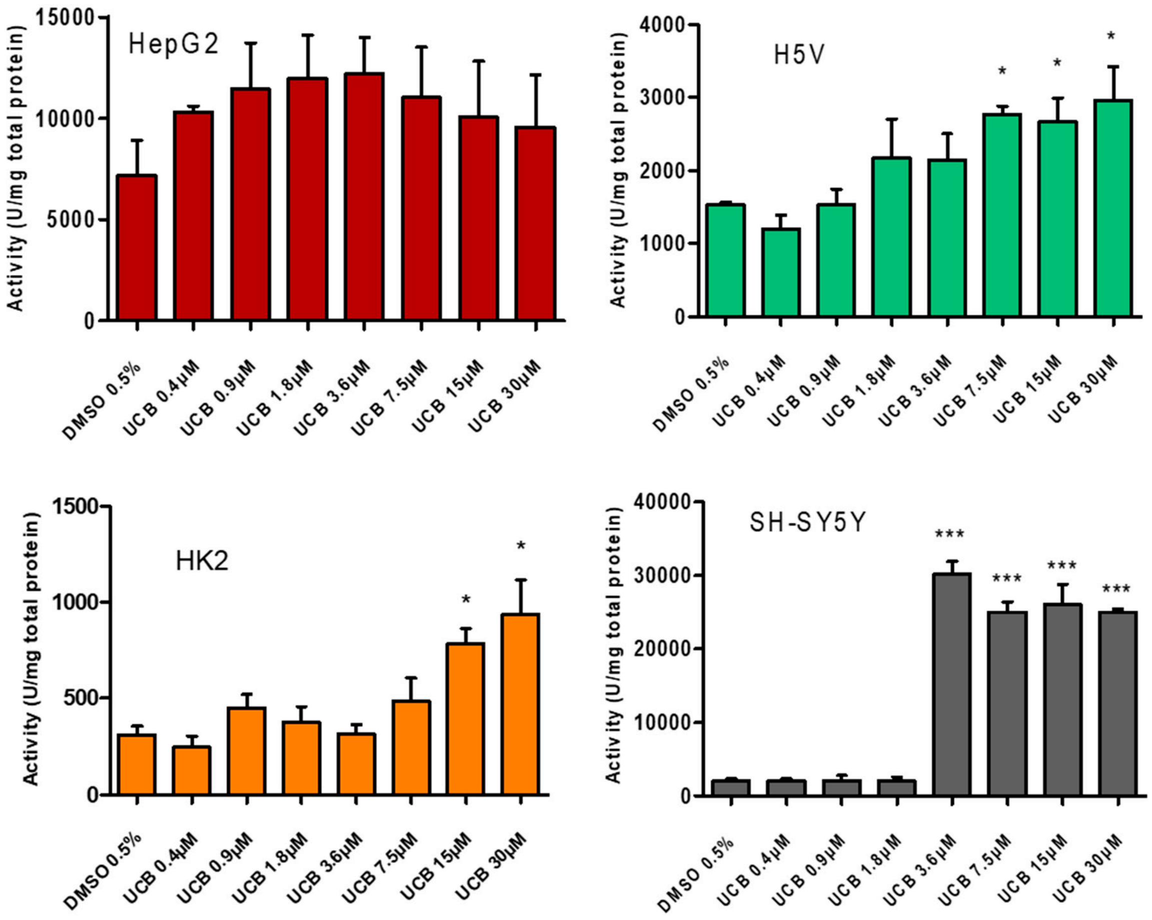

Figure S2. The effect of UCB exposure on SOD activity. The cell lines were exposed to the increasing UCB concentrations (from 0.4 to 30 µM in the presence of 30 µM BSA) or 0.5% DMSO for 24 h and then SOD activity was measured. Data are expressed as mean±SD of three independent experiments. *p<0.05, **p<0.01, ***p<0.001.
